# Unilateral Reconstruction of the Large Breast: Combining Prosthetic and Autologous Methods for Improved Symmetry

**DOI:** 10.1097/GOX.0000000000002154

**Published:** 2019-02-11

**Authors:** Jean-Claude Schwartz

**Affiliations:** From Georgia Breast Surgery, PC, Lawrenceville, Ga.

## Abstract

Postmastectomy reconstruction in patients with significant macromastia and/or large chest wall dimensions can be challenging. Implants have a limited size range and may not be large enough to adequately reconstruct a wide, obese patient. Abdominally based flaps may be unsafe in these patients if they have significant obesity and or other comorbidities and may still be insufficient to adequately fill a very large breast footprint. These problems are compounded in the patient who is not a candidate for an abdominal flap and who undergoes unilateral mastectomy as the contralateral breast, even after aggressive reduction, may still require volume and dimensions that cannot be easily replicated with prosthetic methods alone. Therefore, it seems reasonable to supplement our implant-based reconstructions with additional autologous tissue to reconstruct the breast after mastectomy to obtain acceptable symmetry with the large, contralateral native breast. Here, we report a case of combining the largest available anatomic implant with an extended lateral intercostal artery perforator flap to reconstruct a large breast and obtain symmetry with the native breast.

Patients with large frames, obesity, and macromastia pose significant challenges to the reconstructive breast surgeon after mastectomy.^1^ In addition to the increased complication rates seen in patients with an elevated body mass index (BMI), implant-based or autologous methods alone may be insufficient to achieve a satisfactory result. The breast footprints of these patients invariably require volumes that are unavailable with standard silicone or saline implants and often cannot be reached with abdominally based flaps alone. Even when sufficient abdominal tissue is available to supply these larger volumes, this approach can have prohibitive complication rates in obese patients.^2^ Achieving a satisfactory reconstruction is even more challenging after a unilateral mastectomy as the footprint of the contralateral healthy breast cannot be completely altered (as can be done after risk-reducing mastectomy), and a minimum volume may be required to reconstruct the affected breast and obtain symmetry.

The breast footprint of the native breast must be replicated when performing the postmastectomy reconstruction, and prosthetic methods alone may be insufficient to create a similar-sized footprint. Here, we present a case where we combined use of the largest anatomical implant available after nipple-sparing mastectomy with the lateral intercostal artery perforator (LICAP) flap in a subsequent surgery to obtain symmetry with the large, contralateral native breast and its associated footprint.

## CASE REPORT

**Fig. 1.**
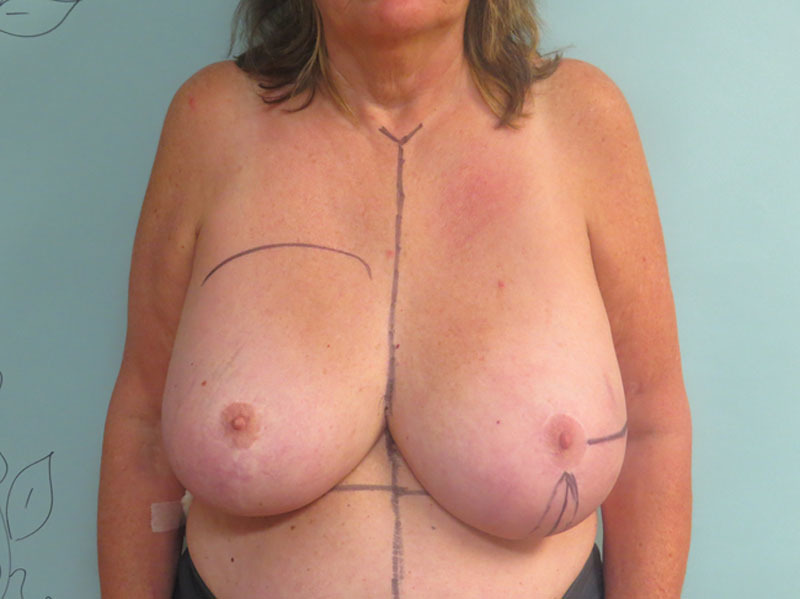
Sixty-three-year-old woman (BMI = 31.8 kg/m^2^) with multicentric left breast cancer requiring mastectomy. She refuses contralateral mastectomy and desires nipple preservation. She has a history of a 1,700 g inferior pedicle breast reduction 20 years ago. She had a significant pseudoptosis with a large nipple to inframammary fold distance, which will be difficult to reconstruct after unilateral mastectomy. We proceed with a nipple-sparing mastectomy through her previous vertical limb and immediate prepectoral breast reconstruction with a full height variable projection tissue expander (width = 16 cm, height = 16.5 cm, and projection =6.8 cm, volume = 850 ml) with complete anterior coverage with an acellular dermal matrix.

**Fig. 2.**
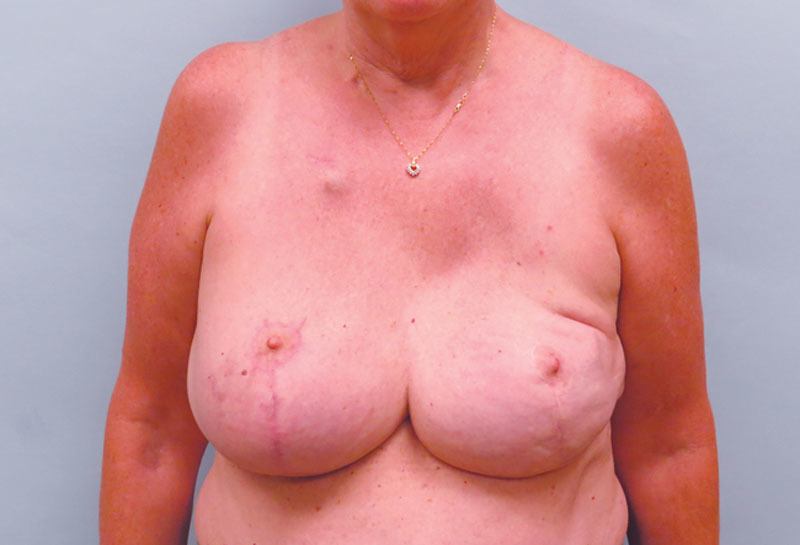
Three months after exchange of her tissue expander for a full height extraprojection anatomic 775-ml implant (width = 15.5 cm, height = 16 cm, and projection = 7.1 cm). She also undergoes a contralateral reduction of 300 g to achieve better symmetry. Although we were able to achieve acceptable symmetry with regards to width and projection by reducing the native breast, we cannot appreciably alter the position of the inframammary fold or the height of the right breast. These landmarks must be respected during the left breast reconstruction and requires a volume that cannot be supplied by an implant alone. She requires additional volume supplementation to reconstruct the upper pole of her left breast.

**Fig. 3.**
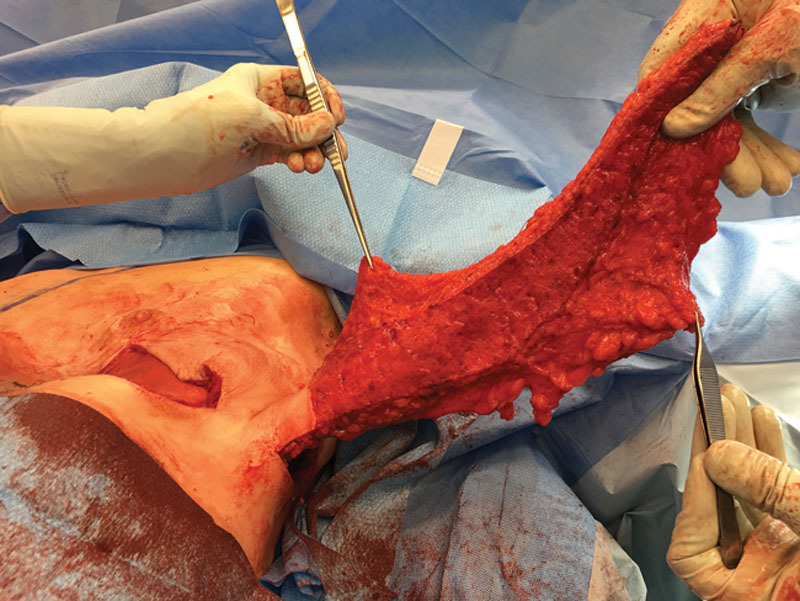
Intraoperative photograph of the patient in the lateral decubitus position with the extended LICAP flap raised and ready for tunneling into the superior pole of the reconstructed breast mound. The flap is secured to the pectoral muscle. We do not violate the capsule around the implant to protect it from any complications that might arise with the flap. A drain is placed at the donor site in the back and underneath the flap anteriorly. An implant sizer is used to optimize flap and implant positioning.

**Fig. 4.**
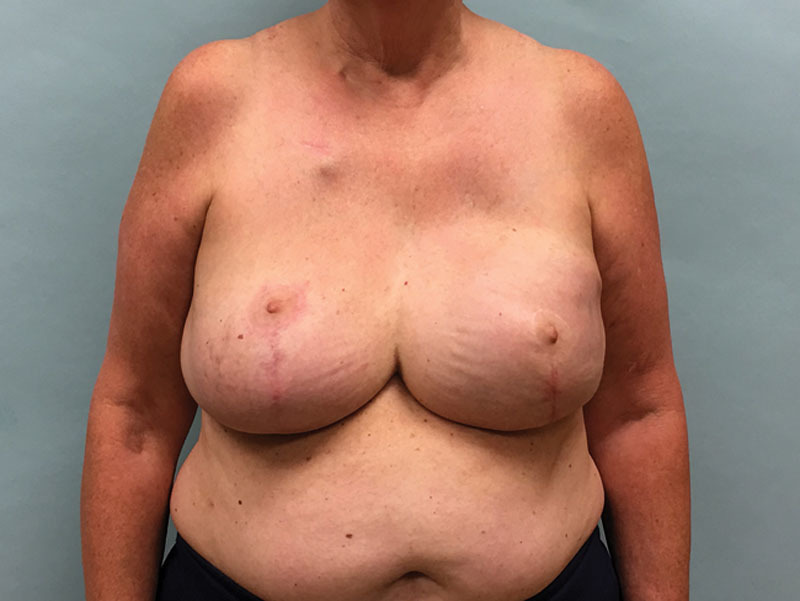
Six months after LICAP supplementation, we have good size symmetry between the breasts, and the upper pole of the left breast has been adequately reconstructed. The right nipple is placed 1–2 cm too high, which detracts from the overall result.

Sixty-three-year-old woman presented with multicentric left breast cancer requiring mastectomy. She is 69 inches tall with a weight of 215 pounds and a corresponding BMI of 31.8 kg/m^2^. She has a history of inferior pedicle breast reduction surgery performed 20 years ago (Fig. 1). Despite her previous reduction, she has a very large breast volume and footprint that will be difficult to replicate on the reconstructed left side. She desires nipple preservation and wants to keep her native right breast. We proceed with a left nipple-sparing mastectomy through her previous vertical limb and immediate prepectoral reconstruction using a full-height variable-projection tissue expander (width = 16 cm, height = 16.5 cm, projection = 6.8 cm, and volume = 850 ml) with anterior coverage using an acellular dermal matrix. Twelve weeks later, we exchange her tissue expander for the largest anatomical implant available, a full-height extra-projection 775 ml implant (width = 15.5 cm, height = 16 cm, and projection = 7.1 cm). She also undergoes a contralateral reduction of 300 g to achieve better symmetry. The final result is shown in Figure 2. Despite using the largest and tallest implant available and reducing the right breast by an additional 300 g, the entire upper pole of the left breast was depleted with significant size asymmetry between the breasts. We discussed multiple sessions of lipofilling to fill this defect but felt that an autologous flap would be more definitive. The LICAP flap was chosen to reconstruct the upper pole of the left breast. An intraoperative photograph is shown in Figure 3, where an extended flap is dissected based off the known perforators that arise anterior to the latissimus muscle at the level of the inframammary fold as previously described.^3^ This flap is rotated on its pivot point and used to reconstruct the upper pole of the breast by suturing it to the underlying pectoralis muscle. The final result 6 months after surgery is shown in Figure 4, where we have reconstructed the upper pole of her breast with the LICAP flap and have acceptable symmetry between the 2 sides. The patient is discharged the same day, and drains are removed on postoperative day 4.

## DISCUSSION

The strategy of using both prosthetic and autologous methods for postmastectomy reconstruction is not new. This approach is most commonly used in the setting of postmastectomy radiotherapy where an autologous flap serves to protect the implant from the known high rates of complications that occur after radiotherapy.^4,5^ The use of an implant is also not uncommonly used in conjunction with an abdominally based flap to provide additional volume in patients with insufficient lower abdominal soft tissue.^6^ The use of abdominally based flaps and implants also allow one to obtain qualities from each that would not be available from either strategy alone, with the implant supplying the needed projection and the flap providing natural ptosis.^7^

Seventy to eighty percent of postmastectomy reconstruction in the United States is implant based. In patients with elevated BMIs and or very large thoracic sizes, the biggest implant sizes available may not provide these patients with a reconstructed breast that is of appropriate size and dimension for their body habitus. While one could consider a microvascular abdominally based free flap with or without implant supplementation, this approach may be daunting in the obese patient with other comorbidities. The reconstructive challenge is even more difficult in unilateral reconstructions as the footprint of the healthy breast cannot be completely reset to smaller, more manageable dimensions as can be obtained after prophylactic mastectomy. In unilateral cases, the reconstructed breast must match the footprint of the native breast, which cannot be completely altered. While the volume, width, and projection of the breast may be reduced, breast height and inframammary fold location remain relatively unchanged. In the case presented here, despite using the largest implant available with regards to width, height, and projection, the entire upper pole of the reconstructed breast was depleted. We supplied this additional volume with the LICAP flap in a subsequent third surgery, providing acceptable symmetry with the contralateral breast. Other groups have described the use of 2 implants in combination with the latissimus flap or the use of the an implant to reconstruct the upper pole with the latissimus filling the lower pole to reconstruct the large breast and obtain symmetry in a patient who was a poor candidate for an abdominal flap who chose to undergo unilateral mastectomy and reconstruction.^8,9^

## SUMMARY

The use of the largest available implants in concert with the LICAP flap may provide surgeons with a safe and minimally morbid strategy to obtain acceptable symmetry after unilateral mastectomy and reconstruction in patients with large breast footprints and macromastia. This approach may have advantages over the use of abdominally based flaps with regards to complexity, operative time, donor site complications, recovery, resources, and safety in patients with comorbidities. This approach is also less morbid than the traditional latissimus dorsi flap with a quicker recovery, less pain, shorter hospitalization, no long-term functional deficits, and no significant incidence of donor site seroma.
